# Quality of life in parents of preterm infants in a randomized nutritional intervention trial

**DOI:** 10.3402/fnr.v60.32162

**Published:** 2016-11-11

**Authors:** Trond Nordheim, Tone Rustøen, Per O. Iversen, Britt Nakstad

**Affiliations:** 1 Department of Pediatric and Adolescent Medicine, Akershus University Hospital, Nordbyhagen, Norway; 2 Institute for Clinical Medicine, Campus Ahus, University of Oslo, Nordbyhagen, Norway; 3 Department of Research and Development, Division of Emergencies and Critical Care, Oslo University Hospital, Oslo, Norway; 4 Department of Nursing Science, Institute of Health and Society, University of Oslo, Oslo, Norway; 5 Department of Nutrition, Institute of Basic Medical Sciences, Faculty of Medicine, University of Oslo, Oslo, Norway; 6 Department of Haematology, Oslo University Hospital, Oslo, Norway

**Keywords:** anxiety, depression, parents, premature, quality of life, very low birth weight

## Abstract

**Background:**

Being a parent of a very-low birth weight (VLBW, birth weight <1,500 g) infant is challenging because of the numerous complications these infants may encounter, many of which are caused by inadequate nutrition. Whether the burden to the parents increases when their VLBW infant participates in a randomized intervention trial (RCT) and is thus exposed to additional risk is unknown.

**Objective:**

To examine parental qualify of life (QoL) and well-being after participation of their VLBW infants in a nutrition RCT.

**Design:**

QoL and symptoms associated with well-being of parents of VLBW infants participating in a nutrition RCT (*n*=31) and of a reference group (parents of nonparticipating VBLW infants, *n*=31) were examined. Assessments were performed when their infants were in the neonatal intensive care unit (NICU) (time point T1) and concurrently at 3.5 years of age (time point T2). The parents completed the following questionnaires: Quality of Life Scale, Hospital Anxiety and Depression Scale, Lee Fatigue Scale (LFS), and General Sleeping Disturbance Scale (GSDS).

**Results:**

At T1, the QoL was better among RCT parents (*p*=0.02). At T2, the RCT parents reported less sleep disturbance symptoms (GSDS) (*p*=0.03) and more energy (LFS) (*p*=0.03).

**Conclusion:**

The RCT participation of VLBW infants may have improved parental QoL. While in the neonatal unit, symptoms of anxiety and depression were common among all parents. The high incidence of anxiety and depression in parents must be considered in the care of parents in the NICU. Long-term effects of participation seem to be less sleep problems and more energy.

For most parents, the sudden arrival of a premature infant is unexpected, overwhelming, and frightening. Being a parent of a critically sick newborn has been described as being in another world, alien from what they knew and had experienced earlier (1). Various studies have described the preterm children's health-related quality of life (QoL) (2–5), but less is known about parental QoL over time (6).

Researchers perform randomized studies to improve treatment and outcome of premature infants. The effect that participation may have on parents is unknown. Parents are invited to enroll their sick child in a study that has no guarantee of benefit to the infant. In an interview study, parents reported that they were grateful for the opportunity to participate, pleased that they could help other infants in the future, and believed that the participation could give access to better medical care and technology for their own infant (7). To our knowledge, no study has evaluated symptoms and QoL among parents of very-low birth weight infants (VLBW, birth weight <1,500 g) enrolled in a clinical randomized intervention trial (RCT). If the parents experience low QoL, symptoms of anxiety, depression, worse physical condition, and/or sleep disturbance, this could affect their children negatively. A Finnish study found an association between poor parental well-being and subsequent behavioral and emotional problems in their VLBW children (8), and a meta-analysis on parental depression stated that both parental and maternal depression affected child development (9).

Fifteen million preterm births worldwide yearly is a global health challenge (10). To address the putative importance of an adequate nutritional intake among VLBW infants, we have performed an intervention RCT – the PreNu study – to enhance their nutrient supply of protein and energy (11, 12). The PreNu study was originally powered to halve the proportion of infants discharged as growth restricted. A preplanned safety analysis revealed increased occurrence of late-onset septicemia in the intervention group (11). Further inclusion of infants was therefore stopped, a decision supported by the Regional Committee for Medical and Health Research Ethics.

In the present study, we explored the parent's QoL and selected symptoms – anxiety, depression, sleep disturbance, and fatigue – during their infant's hospital stay and at age 3.5 years. We compared parents in the PreNu study with parents whose VLBW infants never participated in a clinical research trial.

## Subjects and methods

### Parent cohort

Parents of VLBW infants were enrolled in the PreNu study at Akershus and Oslo University hospitals in Norway. Fifty of 57 eligible infants were recruited from August to December 2010 (12). Inclusion criteria were birth weight <1,500 g and born in the study hospitals. Exclusion criteria were congenital malformations, chromosomal abnormalities, critical illness with short life expectancy, and clinical syndromes known to affect growth and development.

In total, 26 eligible PreNu couples represented by 30 single parents at time point 1 (T1) and 31 single parents at time point 2 (T2) were enrolled in our study. For the purposes of the current study, the PreNu intervention and the PreNu control groups are analyzed together as one group denoted as the PreNu group. All infants received the same attention and follow-up by the researchers. Parents of children in foster care and parents who did not master the Norwegian language were not invited. Some were twins (four pairs) and triplets (one set).

A reference cohort of 25 eligible couples represented by 31 single parents whose VLBW infants never participated in a clinical study was randomly recruited with identical inclusion criteria. They were recruited at the study hospitals during the 3 months before and 3 months after the PreNu inclusion period by a neonatologist not involved in the PreNu study.

### Time points of data collection

Data for the hospitalized period was collected retrospectively 3 years after birth (T1). The follow-up study of parental well-being was conceived in 2014, that is, 3.5 years after the inclusion period of the PreNu study (T2). We analyzed measures of self-report in the PreNu parent group and compared these measures with those of the reference group, both for their in the neonatal intensive care unit (NICU) period and at age 3.5 years. The T1 measures came with a clear instruction to answer as they would have done at the time their infant was in the NICU, and the items of the measures were phrased in ‘past tense’. In the follow-up (T2), parents were invited to complete a new set of questionnaire.

### Data collection

In addition to demographic parental characteristics, these questionnaires were used to collect data about quality of life (QoL) and symptoms in parents.

Quality of Life Scale-Norwegian version (QoLS-N) was used to measure QoL (13, 14). The QoLS-N consists of 18 items to which respondents rate their satisfaction with various aspects of life on a 9-point scale from 1 to 9. A higher score indicates better QoL. This scale is found to have solid psychometric properties, validity and reliability are well established, and the scale is commonly used in the Norwegian population. As a measure of internal consistency and reliability, we used Cronbach's alpha; in the present study, this value was 0.91 for the total QoLS-N scale.

Hospital Anxiety and Depression Scale (HADS) was used to measure anxiety and depression (15). HADS consists of 14 items: 7 measure anxiety and 7 measure depression on a scale from 0 (not at all) to 3 (very much). A cut-off of 8 points is identified for both anxiety and depression (16): mild (8–10), moderate (11–14), and severe (15–21, 15). HADS has been widely used and shows satisfactory psychometric properties for somatic, psychiatric, and primary care patients and in the general population, including Norwegian samples (17). The Cronbach's alpha was 0.80 for anxiety and 0.84 for depression in the present study.

General Sleep Disturbance Scale (GSDS) uses 21 items that measure different aspects in relation to sleep: quality, awakening, and rhythm. Each question ranges from 0 (never) to 7 (every day) on a numeric rating scale. Each of the individual scores can be added to a total score from 0 (no disturbance) to 147 (extremely disturbed) (18). The cut-off point for sleep disturbance versus normal sleep is established at 43 points. Validity and reliability are well established in many patient populations and in healthy individuals, including Norwegian patients with pulmonary disease (19). The Cronbach's alpha was 0.73 in the present study.

Lee Fatigue Scale (LFS) was used to measure fatigue (20). LFS consists of 18 items and two subscales: fatigue and energy. Severity score is documented on both scales from 0 to 10, where higher scores indicate more fatigue. LFS is used in a diversity of populations and also in healthy individuals (20). Validity and internal consistency are well established, and the instrument has been applied to many Norwegian samples and shows satisfactory psychometric properties (21). In the present study, Cronbach's alpha was 0.94 for LFS fatigue and 0.85 for LFS energy.

Self-Administered Comorbidity Questionnaire (SCQ) (22) was used to measure comorbidity. SCQ explores presence of disease, treatment, and whether the disease limits level of activity. The questionnaire is used in different patients groups, including Norwegians (23).

Stressful Likert Scale was used to classify and grade how stressful PreNu parents found the PreNu study participation, on a scale ranging from 1 (not stressful at all) to 5 (very stressful).

### Statistical analyses

The software program IBM SPSS 22 (IBM Corp., Armonk, NY) was used to analyze all data. Normality of the data was tested visually by Q-Q plots. Mean scores and standard deviations were calculated for measure-indices. Missing items were handled according to the recommended method for each individual questionnaire. We used *t*-tests to evaluate differences in mean scores between groups for normally distributed data. The significance level was set at 0.05.

### Approvals

The PreNu study and this follow-up study were both approved by the Regional Committee for Medical and Health Research Ethics. The PreNu study was registered in Clinical Trials with identifier NCT 01103219.

## Results

### Characteristics of the two-parent cohorts and their children

Thirty-one PreNu parents and 31 reference parents, both fathers and mothers, gave their informed consent to participate. There were 51 families at T1 and 35 accepted to participate in the follow-up (T2); response rates at T2 were 69 and 68%, respectively for the groups. Parents in the PreNu group were significantly older than the parents in the reference group (T1, *p*=0.04; T2, *p*<0.01) (Table 1). At T2, but not at T1, the PreNu group had more additional children than the reference group (*p*=0.02) (Table 1).

**Table 1:** Demographics and clinical characteristics of participants at time points 1 and 2

	Time point 1			Time point 2		
						
	PreNu (*n*=30) (%)	Reference (*n*=31) (%)	*p*	PreNu (*n =*31) (%)	Reference (*n*=28) (%)	*p*
Male sex	43.3	51.5	0.52	45.2	50.0	0.72
Lives with other parent	96.7	100	0.31	93.5	96.4	0.66
Have additional children	56.7	32.3	0.06	61.3	37.0	0.07
	Mean (SD)	Mean (SD)	*p*	Mean (SD)	Mean (SD)	*p*
No. of children/family	1.67 (0.7)	1.45 (0.6)	0.21	1.71 (0.7)	1.35 (0.5)	0.02
Age (years)	34.9 (5.6)	31.7 (5.9)	<0.01	34.8 (5.5)	29.8 (5.4)	<0.01
Comorbidity (SCQ score)	1.53 (2.7)	1.74 (2.5)	0.26	0.55 (1.1)	0.61 (1.4)	0.90
						
Infant characteristics:						
GA (weeks)	28.4 (2.7)	29.7 (2.9)	0.08	28.8 (3.0)	29.5 (3.2)	0.40
Birth weight (g)	1,045 (248)	1,169 (259)	0.06	1,044 (244)	1,128 (270)	0.22

SCQ, Self-Administered Comorbidity Questionnaire; GA, gestational age.

### QoL and symptom burden at T1

At T1, there was a significant difference in QoL between parents in the PreNu group compared with the reference group, with better QoL scores for the PreNu parents (Table 2). No significant differences were found between the two groups for anxiety or depression, or in fatigue, energy, or sleep disturbances. However, compared with the general population, parents in these premature cohorts had scores above the clinical cut-offs (≥8) for both anxiety (50.8; 43.3 and 58.1% of the PreNu and reference parents, respectively) and depression (48.4%; no difference between PreNu and reference parents) at T1 (Table 3).

**Table 2:** Differences in scores between parents in the PreNu group compared to the reference group at time points 1 and 2

	Score at time point 1				Score at time point 2			
		
	PreNu (*n=*30)	Reference (*n=*31)			PreNu (*n=*31)	Reference (*n=*28)		
			
	Mean (SD)	Mean (SD)	*p*	95% CI	Mean (SD)	Mean (SD)	*p*	95% CI
QoLS	85.3 (12.6)	76.9 (2.6)	0.02	1.5 to 15.3	88.8 (11.6)	83.1 (12.8)	0.08	−0.7 to 12.1
HADS depression	6.3 (4.1)	7.8 (5.3)	0.23	−3.9 to 1.0	2.4 (2.6)	2.8 (3.2)	0.61	−2.0 to 1.2
HADS anxiety	7.9 (3.5)	8.9 (4.4)	0.34	−3.0 to 1.0	3.3 (2.6)	5.1 (4.6)	0.07	−3.7 to 0.1
HADS (total)	14.2 (6.7)	16.7 (9.0)	0.23	−6.5 to 1.6	5.7 (5.1)	7.9 (7.2)	0.62	−5.5 to 1.0
GSDS	35.8 (12.0)	40.2 (19.9)	0.30	−12.9 to 4.0	27.1 (8.0)	32.2 (8.8)	0.03	−9.7 to −0.4
LFS fatigue	3.3 (1.8)	3.9 (1.8)	0.20	−1.7 to 0.4	2.4 (1.8)	2.7 (2.1)	0.61	−1.3 to 0.8
LFS energy	4.3 (1.6)	3.6 (2.4)	0.14	−0.2 to 1.5	6.2 (1.4)	5.3 (1.8)	0.03	0.1 to 1.8

CI, confidence interval of the difference between the two groups; QoLS, Quality of Life Scale; HADS, Hospital and Anxiety Depression Scale; GSDS, General Sleep Disturbance Scale; LFS, Lee Fatigue Scale.

**Table 3:** Differences between parents in the PreNu group compared to the reference group on level of anxiety and depression at time points 1 and 2

		Time point 1			Time point 2		
			
		PreNu (*n=*30)	Reference (*n=*31)	Total	PreNu (*n=*31)	Reference (*n=*28)	Total
			
		% (*n*)	% (*n*)	% (*n*)	% (*n*)	% (*n*)	% (*n*)
HADS	Normal	56.7 (17)	41.9 (13)	49.2 (30)	93.5 (29)	78.6 (22)	86.4 (51)
anxiety	Mild	20 (6)	19.4 (6)	19.7 (12)	6.5 (2)	10.7 (3)	8.5 (5)
	Moderate	20 (6)	29 (9)	24.6 (15)	0	3.6 (1)	1.7 (1)
	Severe	3.3 (1)	9.7 (3)	6.6 (4)	0	7.1 (2)	3.4 (2)
HADS	Normal	60 (18)	51.6 (16)	55.7 (34)	96.8 (30)	92.9 (26)	94.9 (56)
depression	Mild	26.7 (8)	19.4 (6)	23 (14)	0	0	0
	Moderate	10 (3)	16.1 (5)	13.1 (8)	3.2 (1)	7.1 (2)	5.1 (3)
	Severe	3.3 (1)	12.9 (4)	8.2 (5)	0	0	0

HADS, Hospital and Anxiety Depression Scale.

### Changes in symptom scores from T1 to T2

There was a significant improvement in anxiety, depression, sleep disturbance, fatigue, and energy for both groups of parents pooled between T1 and T2 (Table 4).

**Table 4:** Differences on anxiety, depression, sleep disturbances, and fatigue at time points 1 and 2

	Time point 1				Time point 2				Difference between the two time points				
			
	Mean	*n*	Range	SD	Mean	*N*	Range	SD	Mean	SD	*N*	*P*	95% CI
HADS anxiety	8.4	61	0 to 17	4.0	4.2	59	0 to 20	3.8	4.1	3.7	45	<0.01	3.0 to 5.2
HADS depression	7.0	61	0 to 18	4.8	2.6	59	0 to 13	3.0	4.5	4.1	45	<0.01	3.2 to 5.7
HADS total	15.5	61	1 to 35	8.0	6.7	59	0 to 32	6.3	8.6	6.2	45	<0.01	6.7 to 10.4
GSDS	38.0	61	0 to 76	16.5	29.4	53	13 to 56	8.7	7.9	14.5	42	<0.01	3.4 to 12.4
QoLS	81	61	40 to 109	14.0	86.2	58	59 to 112	12.4	−3.6	12.3	45	0.05	−7.3 to 0.6
LFS fatigue	3.6	61	0 to 8.2	2.0	2.5	59	0 to 7.9	1.9	1.1	1.8	45	<0.01	−0.5 to 1.6
LFS energy	4.0	61	0.2 to 8.0	1.7	5.8	59	1.6 to 9.6	1.7	2.2	2.2	45	<0.01	−2.5 to 5.6

Data are from both study groups pooled. CI, confidence interval of the difference between the time points; HADS, Hospital and Anxiety Depression Scale; GSDS, General Sleep Disturbance Scale; QoLS, Quality of Life Scale; LFS, Lee Fatigue Scale.

### QoL and symptom burden at T2

There was no significant difference between the PreNu and the reference group at T2 regarding QoL, anxiety, depression, or fatigue, but the PreNu group reported significantly more energy and less sleep disturbance at T2 (Table 2). At T2, only 13.6% of all parents scored above cut-off (≥8) for anxiety and 5.1% for depression (Table 3).

### Stress related to participation in PreNu study

Twenty-six percent of the PreNu parents found their infant's participation in the PreNu study to be mildly or a little stressful. None rated participation to be clearly or very stressful. Those who reported participation to be stressful experienced significantly more anxiety and sleep disturbance at T1, but not at T2 (Table 5).

**Table 5:** Levels of anxiety, depression, and sleep disturbance related to level of stress among the PreNu parents

	Stressful (*n*=8)	Not stressful (*n*=22)		
			
	Mean (SD)	Mean (SD)	*p*	95% CI
HADS anxiety	11 (3.3)	6.8 (2.8)	<0.01	1.7 to 6.7
HADS depression	8.1 (2.6)	5.6 (4.4)	0.15	−0.9 to 5.9
HADS total	19.1 (5.2)	12.4 (6.3)	0.01	−1.6 to 11.8
GSDS	43.8 (11.9)	22.9 (10.8)	0.03	1.5 to 20.3

CI, confidence interval of the difference between those who thought participation in the randomized intervention trial was stressful and those who did not think it was stressful; HADS, Hospital and Depression Scale; GSDS, General Sleep Disturbance Scale.

## Discussion

Parents of VLBW infants enrolled in the nutrition RCT PreNu trial reported significantly better QoL compared to reference parents during participation in the trial while hospitalized in the NICU. The reference group reported QoL scores similar to those of unemployed men and women (mean, 74.6; SD, 16.8) and individuals with various health problems (mean, 79.6; SD, 13.8) in the general Norwegian population (14). At T1, the parent PreNu group had higher QoL scores than comparable age groups in the general population (mean score for 30–39 years, 83.1; SD, 12.5) (14). At T2, the QoL in both parent groups had improved, with mean scores similar to those in the general population (14).

The difference in QoL between the PreNu parents and the reference group may be explained by a closer follow-up, more time spent in conversations, and interest shown by the research group members. The effect of the amount of follow-up on positive outcome is in accordance with other studies (24). Extensive contact with relevant health professionals during a hospital stay is shown to be beneficial for adults and can reduce symptoms of anxiety and depression (25).

One fourth of the PreNu parents thought participating in the trial was stressful, even though they scored better on QoL than those whose infant never participated in a RCT. This ‘stressed’ group had more symptoms of anxiety and sleep disturbance compared to the ‘nonstressed’ group. Being a VLBW infant parent is stressful, and it is difficult for the parent to evaluate the possible additive stress effect due to participation in an RCT. Cartwright et al. (7) found that parental stress is caused by having a sick infant in the NICU rather than by participating in the RCT. We found symptoms of anxiety in both in the PreNu and the reference group, which may impact on the reported stress (26).

QoL may affect a parent's ability to accept his or her infant and be sensitive to that infant's needs. Adverse life conditions and symptom burden in parents and caregivers may result in suboptimal nurture of their infant (8). It is important to explore whether participation in an intervention study can negatively impact on the burden of stress, and whether this impact is within the ethical standards for clinical research. We found no significant difference between the PreNu parents and reference group parents regarding anxiety and depression. This observation is important because severe maternal depression has been associated with poor child developmental outcome, including in VLBW infants (9). Not surprisingly, symptoms of anxiety and depression were very common in both parent groups while the infant was hospitalized. This finding can be attributed to the experience of becoming parents to a sick prematurely born infant. Clinical staff need to be aware of this and should offer support to parents in this vulnerable situation.

The PreNu parents reported less sleeping disturbance and were more energetic than the reference group 3.5 years after the birth of their child (T2). One speculation could be that a higher QoL and satisfaction with life at the time the infants were hospitalized could be protective for sleeping problems. Sleep is important to health and recovery from illness, and postpartum psychological reactions and quality of sleep are related problems (27).

Parents in the PreNu group were slightly older than those in the reference group. Advancing age may be associated with lower QoL (14); however, in the present study, parents in the PreNu group were older and had higher QoL. Adjusting for age had no effect on the QoL. At T2, the parents in the PreNu group had significantly more children than the reference parents. In spite of having more children, the PreNu group had less sleep disturbance and more energy than the reference group.

Several considerations may explain the positive effect of attending an RCT. A Danish study of the effect of supportive communication on parental stress in a NICU found no effect on parental stress, but it raised concern about the need to support parents also in periods where the infant's clinical condition was stable and around discharge from the NICU (28). In our study, PreNu parents were closely followed from birth until 52 weeks age, several weeks after discharge. In addition comes the plausible benefit of providing the infant with a putative better nutrition regime.

The main motivating factor for consenting to their child's participation in an RCT is a belief in a direct benefit for the child (29). Parents may have felt more secure that their infant received the best possible treatment and thus relaxed more, despite the fact that the spoken and written invitation to PreNu was neutral, emphasizing that we did not know whether the intervention was good or bad for the infant. In an interview study focusing on parents’ perceptions of their infants’ participation in an RCT (7), parents thought their infants were given the best possible care because of their participation. Furthermore, the parents appreciated being given the choice of consent, thus putting them in a situation where they have the perception of being in control. At the core of the concept of sense of coherence (SOC) are the individual's experiences of comprehensibility, meaningfulness, and manageability of one's life (8). SOC is shown to be related to the psychosocial effects of health problems. Consenting may make the parents feel that they do something good for their infants and that they are actively exercising parental authority. Participation in an RCT could make the parents feel that their stay in the NICU is more purposeful.

The small number of respondents and the retrospective design at T1 are the main limitations in our study. The number of parents in the present study was determined by the number of participating children in the PreNu study. Because this study was planned after the PreNu recruitment period, we could only assess the parent situation at T1 retrospectively, and the quantitative information of the parent's well-being in this period was therefore retrospective. Both groups of parents had infants hospitalized in the same hospitals with the same routines and staff. To avoid bias, we invited and enrolled parents from the immediate period before and after the PreNu trial was run in these neonatal units. The two study groups were comparable regarding sex, comorbidity, the birth weight, and gestational age, and we did control for age of parent and number of children. The PreNu parents may be a selected group simply because of their sustained participation in the trial. In general, pediatric trials obtain a high participation rate (30), and this trial is no exception. Seventy percent of all eligible parents were recruited, implying that the PreNu parents are a representative group of VLBW parents.

Retrospective assessment may not be accurate, making it difficult to generalize and compare with prospective studies with the same measures. Self-report is by nature subjective and often makes the subject view his or her situation retrospectively. We believe that the experience of becoming a parent of a VLBW infant is remembered very vividly because of the emotional salience. A strength of this study is that our outcome measures had excellent internal consistency for both the retrospectively assessed T1 and the concurrently assessed T2 (with the exception of sleep disturbance).

It was not possible to control every psychosocial variable related to well-being, and correcting *p* values for multiple testing may have decreased the likelihood of false positives. However, because of the small size of our groups, it is unlikely that any clear and relevant difference would have been proven statistically significant on correction for multiple testing. Nevertheless, we believe that our extensive battery of measurement tools, the logical consistence of the findings, and relationships between the measures add credence to our results.

## Conclusions

Parents of an infant born VLBW who participated in the PreNu intervention RCT experienced better QoL when the infant was hospitalized, compared to a reference group. Symptoms of anxiety and depression affected half of the parents, but this number improved and was normalized when their infant was 3.5 years of age. The high incidence of anxiety and depression in parents must be considered in the care of parents in the NICU. Long-term effects of participation seem to be less sleep problems and more energy. In recruiting newborn VLBW infants for an RCT trial, researchers can most likely be confident that participation in itself is not a burden for the parents and thus does not constitute an unacceptable risk for the infant. Parents in an RCT trial may feel more secure because of extra follow-up, but this hypothesis warrants more research.
